# Machine learning prognostication in nasopharyngeal carcinoma: a european multicentre analysis of survival and risk of second malignancy

**DOI:** 10.1007/s00405-026-10461-z

**Published:** 2026-07-17

**Authors:** Irina Maria Pușcaș, Anda Gata, Paolo Boscolo Rizzo, Marco Stellin, Vittorio Rampinelli, Davide Tomasini, Cesare Piazza, Daniele Borsetto, Will Ince, Carlos M. Chiesa-Estomba, Maria Landa-Garmendia, Pavol Surda, Eleanor Crossley, Matt Lechner, Teodora Maria Ursu, Laura Diana Cernău, Adél Bajcsi, Laura Silvia Diosan, Camelia Chira, Alexandra Roman, Vlad Alexandru Gâta, Alexandru Irimie, Silviu Albu

**Affiliations:** 1 Department of Paediatric Otorhinolaryngology, “Iuliu Hatieganu” University of Medicine and Pharmacy, Cluj-Napoca, Romania; 2 Department of Otorhinolaryngology, “Iuliu Hatieganu” University of Medicine and Pharmacy, Cluj Napoca, Romania; 3 Department of Neurosciences, University of Padova, Treviso, Italy; 4 Department of Medical, Surgical and Health Sciences, Section of Otolaryngology, University of Trieste, Trieste, Italy; 5 Unit of Otorhinolaryngology, Head and Neck Surgery, Department of Surgical Specialties, Radiological Sciences and Public Health, University of Brescia, Brescia, Italy; 6 Unit of Otorhinolaryngology-Head and Neck Surgery, ASST Spedali Civili of Brescia, Brescia, Italy; 7 Department of Medical and Surgical Specialties, Radiological Sciences, and Public Health, School of Medicine, University of Brescia, Brescia, Italy; 8 Department of Otolaryngology - Head & Neck Surgery, Cambridge University Hospitals NHS Trust, Cambridge, United Kingdom; 9 Department of Oncology, Cambridge University Hospitals NHS Trust, Cambridge, United Kingdom; 10 Department of Otolaryngology - Head and Neck Surgery, Donostia University Hospital, Deusto University - Faculty of Medicine, San Sebastián, Bilbao Spain; 11 Department of ENT Surgery, Guy’s and St Thomas’ University Hospital, London, UK; 12 Division of Surgery and Interventional Science and UCL Cancer Institute, University College London, London, UK; 13 Department of Computer Science, Faculty of Mathematics and Computer Science, Babes-Bolyai University, Cluj-Napoca, Romania; 14 Department of Periodontology, “Iuliu Hațieganu” University of Medicine and Pharmacy, Cluj-Napoca, 400012 Romania; 15 Surgical Oncology Department, “Iuliu Hatieganu” University of Medicine and Pharmacy, Cluj-Napoca, Romania

**Keywords:** Nasopharyngeal carcinoma, Non-endemic population, Machine learning, Multicenter European study, Systemic inflammatory markers

## Abstract

**Introduction:**

Nasopharyngeal carcinoma (NPC) is rare in Europe, and emerging data suggest poorer outcomes in Caucasian patients compared with Asian populations, highlighting the need for region-specific prognostic tools. Inflammation-based biomarkers and artificial intelligence show promise for risk stratification and prediction of survival and second primary cancers (SPCs).

**Materials and methods:**

We conducted a retrospective multicentre study including 405 NPC patients from six European institutions. Demographic, clinicopathological, and haematologic inflammatory markers were collected, and machine learning algorithms were developed to predict 5-year OS and SPC occurrence. Multiple train–test splitting strategies and machine learning (ML) classifiers were evaluated. Models were tested both with and without systemic inflammatory ratios to assess their added prognostic value.

**Results:**

The median age was 52 years, 91.6% of patients were classified as White/European ancestry, and 77.3% received chemoradiotherapy. Five-year OS was 66.6%, while 12.8% developed SPC. The Random Forest classifier achieved the best performance for OS prediction (accuracy 0.74; AUC 0.66) using the complete feature set, while SPC prediction reached an accuracy of 0.80 (AUC 0.74). Exclusion of inflammatory markers resulted in a consistent decline in accuracy across all models. Feature-importance analysis highlighted inflammatory ratios among the strongest predictors for both OS and SPC. The present study was reported according to TRIPOD+AI reporting guidelines.

**Conclusions:**

This study presents the first machine-learning prognostic models for nasopharyngeal carcinoma derived from a predominantly Caucasian European multicentre cohort. Systemic inflammatory markers modestly improved overall survival prediction and substantially enhanced second primary cancer risk estimation. The resulting models are transparent, cost-effective, and support the potential benefit of prognostic assessment through machine learning in non-endemic settings.

**Supplementary Information:**

The online version contains supplementary material available at https://doi.org/10.1007/s00405-026-10461-z.

## Introduction

Nasopharyngeal carcinoma (NPC) is a distinct malignancy with marked geographic and ethnic variability, showing highest incidence in Southern China, Southeast Asia, and parts of Africa and the Arctic region [1–3]. Its multifactorial pathogenesis involves Epstein–Barr virus infection, genetic susceptibility, and environmental exposures such as preserved food intake and tobacco use [4]. Although rare in Western populations, emerging evidence suggests that Caucasian patients may experience poorer survival compared with Asian cohorts, underscoring the need for region-specific prognostic strategies [2, 5]. Even though this disparity may have several explanations (different risk factors, distinct EBV biology, different acces to high-volume centers, etc.), it underscores the clinical relevance of NPC even in non-endemic settings and highlights the need for tailored diagnostic, prognostic, and therapeutic approaches for European and other non-Asian patient populations. Moreover, the global incidence of NPC is projected to increase substantially by 2040, reinforcing its growing clinical relevance beyond endemic regions [6]. Despite advances in imaging and treatment modalities, recurrence and distant metastases remain major challenges, and significant outcome heterogeneity persists among patients with similar TNM stages [7–10].

Inflammation is a recognized hallmark of cancer, influencing tumour initiation, progression, angiogenesis, and immune evasion [11]. In NPC, the tumour microenvironment reflects complex interactions between malignant epithelial cells and immune components, including lymphocytes, macrophages, and neutrophils, which may either promote or inhibit tumour progression [3]. Circulating inflammatory indices derived from routine haematologic tests, such as the neutrophil-to-lymphocyte ratio (NLR), platelet-to-lymphocyte ratio (PLR), lymphocyte-to-monocyte ratio (LMR), systemic immune-inflammation index (SII), and systemic inflammation response index (SIRI), reflect the balance between host immune function and tumour-promoting inflammation [9]. These easily measurable, cost-effective biomarkers have shown prognostic value in various malignancies, including NPC [3]. In parallel, artificial intelligence (AI), particularly machine learning (ML), has emerged as a powerful tool capable of modeling complex, nonlinear relationships within oncologic datasets, with promising results in NPC outcome prediction [10, 12–14].

As treatment outcomes continue to improve, survivorship-related complications, particularly second primary cancers (SPCs), have gained increasing clinical relevance. SPCs are a major cause of late mortality in patients with head and neck malignancies, including NPC, but remain less extensively studied in this subgroup [4, 15]. Although intensity-modulated radiotherapy (IMRT) has improved local control and reduced toxicity, concerns persist regarding radiation-associated malignancies due to increased low-dose exposure of surrounding tissues [16]. Given the younger age at diagnosis and prolonged survival of NPC patients, predictive modeling of SPC risk is essential for optimizing long-term surveillance strategies [17].

The low incidence of nasopharyngeal carcinoma in Europe precludes individual centres from independently generating sufficiently large datasets for robust modelling. This study aimed to apply established machine learning methods to develop prognostic models for 5-year overall survival (OS) and SPCs in a non-endemic Caucasian nasopharyngeal carcinoma population, using multicentric European demographic, tumour-related, treatment-related, and pre-treatment inflammatory variables.

## Materials and methods

### Study population

This study was approved by the Ethics Committee of The University of Medicine and Pharmacy ‘Iuliu Hațieganu’ (56/10.03.2021) and each centre participating in the research also obtained Ethics Committee approval before reviewing patient records. The current study is a multicentric collaboration which included patients diagnosed with NPC from six different European centres, including the Oncological Institute of Cluj-Napoca in Romania, University of Treviso and University of Brescia in Italy, University of Donostia San-Sebastian in Spain, Cambridge University and Guy’s and St Thomas’ Hospital in the United Kingdom. Retrospective gathering of data included demographic characteristics, smoking status, medical history, pre-treatment haematologic test results, tumour staging, histology and immunohistochemistry, Epstein Barr virus DNA detection, TNM grading, treatment regimen, post-treatment complications, 5-year overall survival (OS) and the advent of second malignancy during follow-up. Ethnicity data were retrospectively extracted from institutional medical records from the participating centres. Because ethnicity classification systems were not fully standardised across institutions, these variables were harmonised during preprocessing and should be interpreted cautiously.

The inclusion criteria were as follows: (1) histologically confirmed diagnosis of NPC, (2) the selected histology subtypes in patients according to World Health Organization (WHO), included keratinizing squamous cell carcinoma (WHO I), differentiated non-keratinizing carcinoma (WHO II), undifferentiated non-keratinizing carcinoma (WHO III) with a minimum follow-up of 5 years; (3) patients treated with IMRT alone or associated with chemotherapy.

The exclusion criteria were as follows: (1) concurrent malignancies; (2) improper cardiac, pulmonary, renal, haematologic or hepatic functions, interfering with the therapeutic decision for NPC; (3) patient with systemic infections documented in patient files, or identified on pre-treatment blood tests and (4) incomplete record of haematological indicators or clinical and follow-up data.

## Curation of study variables

Based on pre-treatment blood tests, for each patient, inflammation indicators were calculated using total count (10^9^/L) for each parameter as follows: SIRI = neutrophils × monocytes/lymphocytes; SII = neutrophils × platelets/lymphocytes; NLR = neutrophils/lymphocytes; PLR = platelets/lymphocytes; ELR = eosinophils/lymphocytes; BLR = basophil/lymphocytes; LMR = lymphocytes/monocytes.

To define cut-off values for inflammatory indices, laboratory reference data from patients treated at each centre for non-inflammatory, non-neoplastic conditions were collected.

## Model Development and Evaluation

To ensure consistency across the six participating centres, only variables available at all institutions were retained. Categorical features were harmonised using standardised mapping schemes, and missing or inconsistent entries were coded with placeholders; variables remaining unreliable after preprocessing were excluded. Consequently, all patient records used for modelling were complete and internally consistent, enabling robust downstream analysis. Clinical outcomes were defined as binary targets: 5-year OS and SPC occurrence. All machine-learning experiments used an identical preprocessing and evaluation pipeline to ensure fair and consistent comparison across prediction tasks.

To address potential treatment-related heterogeneity across the multicenter cohort, therapeutic interventions were standardized into binary variables (administered vs. not administered). Treatment protocols were highly standardized: 98% of the patients (n = 397) received radiotherapy, while 77.28% (n = 313) were treated with chemotherapy. By including both radiotherapy and chemotherapy as binary features, the machine learning models were able to adjust for the primary treatment effect regardless of the center of origin. This approach, was further strengthened by specific harmonization measures designed to minimize inter-institutional variance, including the selection of a common feature set shared across all six centers and the implementation of a uniform mapping system for all categorical variables. Furthermore, a rigorous data auditing process was conducted to identify and exclude inconsistent variables. In order to further minimize treatment heterogeneity, we excluded patients who did not receive treatment as per protocol for their NPC stage due to concomitant comorbidities or patient refusal.

For model evaluation, datasets were divided into training (70%) and validation (30%) sets using three splitting strategies: random, stratified, and balanced, to ensure that the prognostic performance was not an artifact of a single data distribution. Random splitting strategy provide a baseline for unbiased partitions, facilitating the evaluation of the models’ generalizability on completely randomized data. Stratified splitting preserved the exact proportions of survival and malignancy events across the subsets, ensuring consistent outcome, and balanced splitting addressed class imbalance through undersampling of the majority class in the training set. This triple verification strategy functions as a rigorous internal validation, demonstrating that the predictive power of the models remains robust across varying class densities.

The initial cohort for Second Primary Malignancy prediction comprised 405 patients, characterized by class imbalance where 341 patients (84.2%) had no SPC event (Class 0) and 64 patients (15.8%) were diagnosed with an SPC (Class 1). To ensure the algorithms were not biased toward the majority class, the balanced splitting strategy utilized majority-class undersampling to create a training environment with a 1:1 ratio, consisting of 45 patients from each class. The performance of this balanced model was then evaluated on a validation set of 122 patients (103 Class 0 and 19 Class 1). In contrast, the random split strategy resulted in a training set of 244 Class 0 and 39 Class 1 cases, with 122 patients reserved for validation (97 Class 0 and 25 Class 1). Finally, the stratified split was designed to preserve the original clinical prevalence across both subsets, yielding a training set of 238 Class 0 and 45 Class 1 cases, while the validation cohort maintained the 15.8% event rate with 103 Class 0 and 19 Class 1 patients. This multi-layered approach to data partitioning allows an assessment of how different training distributions impact the model’s ability to identify high-risk individuals in a real-world clinical population.

To assess potential multicollinearity among the input variables, a pairwise correlation analysis was performed. While strong correlation were observed among certain inflammatory indices, such SIRI, NLR and SII, which is expected given their shared haematological components, correlations between these markers and standard clinical features remained remarkably low. This suggests that the inflammatory ratios provide independent prognostic information not captured by traditional staging systems. (Fig. 5)

The selection of machine learning architectures was guided by the need to balance interpretability with robustness in handling high-dimensional complex clinical data. Decision Trees (DT) were utilized for their high interpretability and transparency, providing a structure that closely mirrors clinical reasoning. Random Forest method was selected for its robustness and ability to mitigate the impact of multicollinearity among correlated inflammatory markers. Through the selection of a random subset of predictors at each split, the algorithm decorrelates individual learners, preventing overlapping biomarkers from biasing the model. Support Vector Machines (SVM) were used due their effectivness in high-dimensional spaces where the number of predictors is relatively large compared to the sample size. SVM’s are resilient to multicollinearity as they identify optimal separating hyperplanes based on the geometric distribution of the data, rather than relying on independent feature coefficients.

Each experiment was conducted using two feature sets: a full dataset including clinical variables and haematological biomarkers, and a reduced dataset excluding all blood-based features. Model performance was assessed using specific metrics for each task, with classification models evaluated using Accuracy (overall correct predictions), Precision (the ability to correctly identify high-risk patients), Recall (the proportion of actual events correctly captured), F1-Score (the harmonic mean of precision and recall), and ROC AUC (Area Under the Receiver Operating Characteristic Curve, the primary metric for assessing discriminatory power between classes) with confidence intervals [18].

## Results

### Baseline patient information

**Fig. 1:**
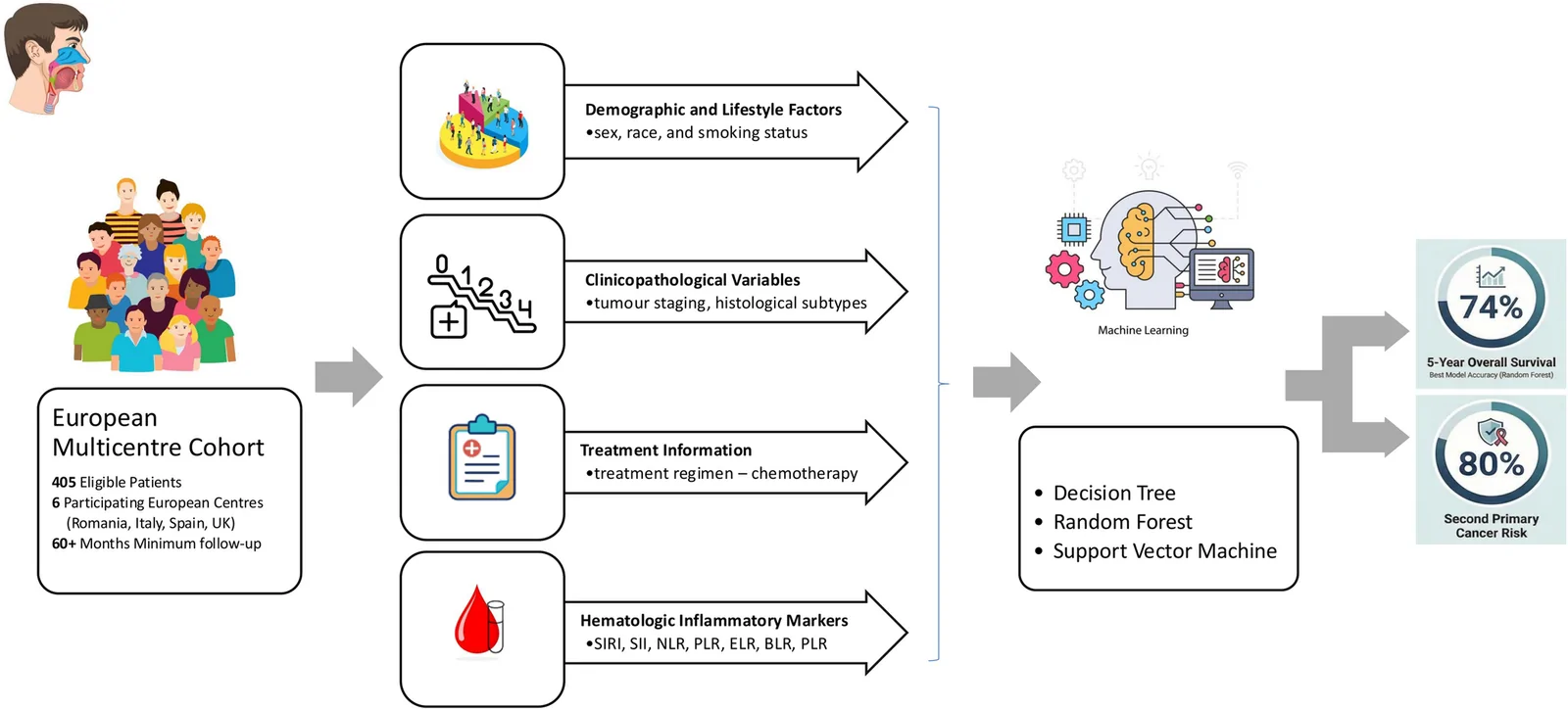
Graphic representation of the multicenter study design workflow

A total of 503 patients were initially identified across six European centres, of whom 405 had complete clinical and laboratory data and were therefore eligible for inclusion in the machine learning analysis. A comparison between the initial database and the final patient population included in the study has been depicted in Table 1, showing similar demographic and tumor-related characteristics of the two populations. The study design workflow is depicted in Fig. 1. The median age across the study population was 52 years (range, 13–78), and 32% of the patients were female. The percentage of smokers was 37.7% across the cohort. Regarding race, 91.6% of patients were classified as White/European ancestry, followed by 2.7% Asian, 1.7% Black, and 3.9% other or mixed ethnic backgrounds. The predominant histological subtype was WHO III, accounting for 81.2% of cases, followed by WHO II at 14.1% and WHO I 4.7%. Most patients presented advanced disease at diagnosis, with T4 stage 32.4%, positive lymph node metastasis (N+) 77.2% and distant metastasis (M+) 3.7%. All patients received IMRT and 77.2% associated chemotherapy in the treatment regimen. 66.6% of patients were alive at 5-year follow-up across the study population and 15.8% of patients developed SPC during follow-up. SPC location included larynx, thyroid, oropharynx, hypopharynx, lung, oesophagus and haematological.

**Table 1:** Comparison of clinicopathological characteristics between the initial study cohort and the final analyzed cohort, following exclusion of patients with incomplete data

Feature	Number of patients after exclusion	Number of patients before exclusion	Percentage after exclusion (%)	Percentage before exclusion (%)
**Sex- male**	275	344	67.9	68.53
**Sex- female**	130	158	32.1	31.47
**Race- White/European**	371	420	91.6	83.67
**Race- Black**	7	26	1.72	5.19
**Race- Asian**	11	22	2.72	4.38
**Race- Other**	16	34	3.96	6.77
**Smoking status- N**	252	324	62.3	64.34
**Smoking status- Y**	153	179	37.7	35.66
**Histology WHO I**	19	19	4.69	3.79
**Histology WHO II**	57	73.0	14.07	14.57
**Histology WHO III**	329	409	81.24	81.64
**T1 stage**	86	142	21.23	28.29
**T2 stage**	107	119	26.42	23.7
**T3 stage**	96	108	23.7	21.51
**T4 stage**	116	133	28.65	26.5
**N0 stage**	92	154	22.72	30.68
**N1 stage**	86	97	21.23	19.32
**N2 stage**	168	183	41.48	36.46
**N 3 stage**	59	68	14.56	13.55
**M0 stage**	390	475	96.3	96.2
**M+ stage**	15	19	3.7	3.8
**Associated chemotherapy**	92	148	22.72	29.48
**Without associated chemotherapy**	313	354	77.28	70.52
**Second Malignancy- absent**	341	434	84.2	86.28
**Second Malignancy-present**	64	69	15.8	13.71

### Machine learning algorithms for prediction

The accuracy of different machine learning algorithms in predicting 5-year OS and SPC are depicted in Tables 2 and 3, respectively. For prediction of 5-year OS in the random distribution of patients between train and validation cohorts, RF classifier that uses all the features achieved best results, with and accuracy in the validation cohort of 0.7459 and AUC: 0.6621. The model utilized an ensemble of 200 individual decision trees, with a maximum depth of 20 levels to capture complex interactions; to ensure structural stability, a minimum of 10 samples was required to split an internal node, while at least 4 samples were maintained per terminal leaf. When we eliminated blood inflammatory markers from the input variables, the algorithm’s accuracy decreased to 0.6967, with AUC: 0.6798.

**Table 2:** Prediction of 5-year OS through three different machine learning algorithms and with the study population split in three manners- stratified, random and balanced- results in validation cohort

Prediction of 5-year OS - Validation								
	Algorithm	Split Type	Accu-racy	Precision	Recall	F1 Score	AUC	95% CI
Algorithms with blood inflammatory markers	Decision Tree	stratified	0,6557	0,6301	0,6557	0,6340	0,5656	[0.45–0.67]
	Random Forest	stratified	0,7049	0,6893	0,7049	0,6685	0,6230	[0.51–0.73]
	SVM	stratified	0,7131	0,7012	0,7131	0,7030	0,6748	[0.57–0.77]
	Decision Tree	random	0,6393	0,6631	0,6393	0,6471	0,5991	[0.5–0.7]
	Random Forest	random	0,7459	0,7616	0,7459	0,7072	0,6621	[0.55–0.77]
	SVM	random	0,6639	0,6509	0,6639	0,6552	0,5933	[0.48–0.7]
	Decision Tree	balanced	0,6885	0,6816	0,6885	0,6843	0,6665	[0.56–0.76]
	Random Forest	balanced	0,6393	0,6580	0,6393	0,6460	0,6076	[0.49–0.72]
	SVM	balanced	0,6967	0,6986	0,6967	0,6976	0,6594	[0.55–0.76]
Algorithms without blood inflammatory markers	Decision Tree	stratified	0,6967	0,6885	0,6967	0,6914	0,6424	[0.54–0.74]
	Random Forest	stratified	0,6885	0,6757	0,6885	0,6790	0,6566	[0.55–0.76]
	SVM	stratified	0,6885	0,6731	0,6885	0,6759	0,6488	[0.54–0.75]
	Decision Tree	random	0,6721	0,6950	0,6721	0,6792	0,6488	[0.54–0.75]
	Random Forest	random	0,6967	0,6916	0,6967	0,6937	0,6798	[0.57–0.78]
	SVM	random	0,6721	0,6550	0,6721	0,6589	0,5905	[0.48–0.71]
	Decision Tree	balanced	0,6721	0,6762	0,6721	0,6740	0,6207	[0.51–0.72]
	Random Forest	balanced	0,6475	0,6635	0,6475	0,6534	0,6361	[0.53–0.74]
	SVM	balanced	0,6803	0,6748	0,6803	0,6772	0,6480	[0.54–0.75]

OS, Overall Survival; SVM, Support Vector Machines; AUC, Area Under the Curve; CI, Confidence Intervals;

**Table 3:** Prediction of SPC through three different machine learning algorithms and with the study population split in three manners- stratified, random and balanced- results in validation cohort

Prediction of SPC - Validation								
	Model	Split Type	Accu-racy	Precision	Recall	F1 Score	AUC	95% CI
Algorithms with blood inflammatory markers	Decision Tree	stratified	0,8279	0,7842	0,8279	0,7972	0,5756	[0.46–0.69]
	Random Forest	stratified	0,8443	0,7128	0,8443	0,7730	0,7154	[0.57–0.85]
	SVM	stratified	0,8197	0,7766	0,8197	0,7916	0,5810	[0.44–0.72]
	Decision Tree	random	0,7787	0,7359	0,7787	0,7468	0,5709	[0.48–0.67]
	Random Forest	random	0,8033	0,8423	0,8033	0,7233	0,7427	[0.63–0.83]
	SVM	random	0,7951	0,7700	0,7951	0,7771	0,2458	[0.16–0.35]
	Decision Tree	balanced	0,6967	0,7754	0,6967	0,7277	0,6035	[0.45–0.75]
	Random Forest	balanced	0,7213	0,8635	0,7213	0,7585	0,7649	[0.62–0.88]
	SVM	balanced	0,8525	0,8275	0,8525	0,8041	0,3470	[0.22–0.5]
Algorithms without blood inflammatory markers	Decision Tree	stratified	0,7951	0,7304	0,7951	0,7585	0,4908	[0.36–0.63]
	Random Forest	stratified	0,8443	0,7128	0,8443	0,7730	0,5552	[0.41–0.71]
	SVM	stratified	0,8361	0,7935	0,8361	0,8027	0,5327	[0.37–0.69]
	Decision Tree	random	0,8033	0,7780	0,8033	0,7360	0,6219	[0.5–0.74]
	Random Forest	random	0,7951	0,6322	0,7951	0,7043	0,6237	[0.51–0.74]
	SVM	random	0,7869	0,7450	0,7869	0,7526	0,5670	[0.43–0.71]
	Decision Tree	balanced	0,6148	0,7338	0,6148	0,6614	0,5202	[0.38–0.66]
	Random Forest	balanced	0,5820	0,7881	0,5820	0,6393	0,5902	[0.45–0.73]
	SVM	balanced	0,5164	0,7365	0,5164	0,5824	0,4870	[0.32–0.64]

SPC, Second Primary Tumour; SVM, Support Vector Machines; AUC, Area Under the Curve; CI, Confidence Intervals

This configuration achieved peak performance using a forest of 50 trees and a maximum depth of 10, implementing a constraint of 10 samples for node splitting and a minimum of 2 samples per leaf. A detailed analysis of the ablation study revealed a nuanced relationship between predictive accuracy and discriminatory power. Upon the exclusion of systemic inflammatory markers, a decline in overall accuracy was observed across all models, confirming that these biomarkers are important for correct patient classification. The marginal increase in the OS AUC from 0.66 to 0.68 in the absence of inflammatory data likely reflects the model’s increased reliance on high-impact anatomical variables like T and N stages. While these standard variables provide strong discrimination for advanced stages, the loss of biomarkers reduces the model’s ability to fine-tune risk for intermediate cases, leading to lower overall accuracy despite a stable AUC.

**Fig. 2:**
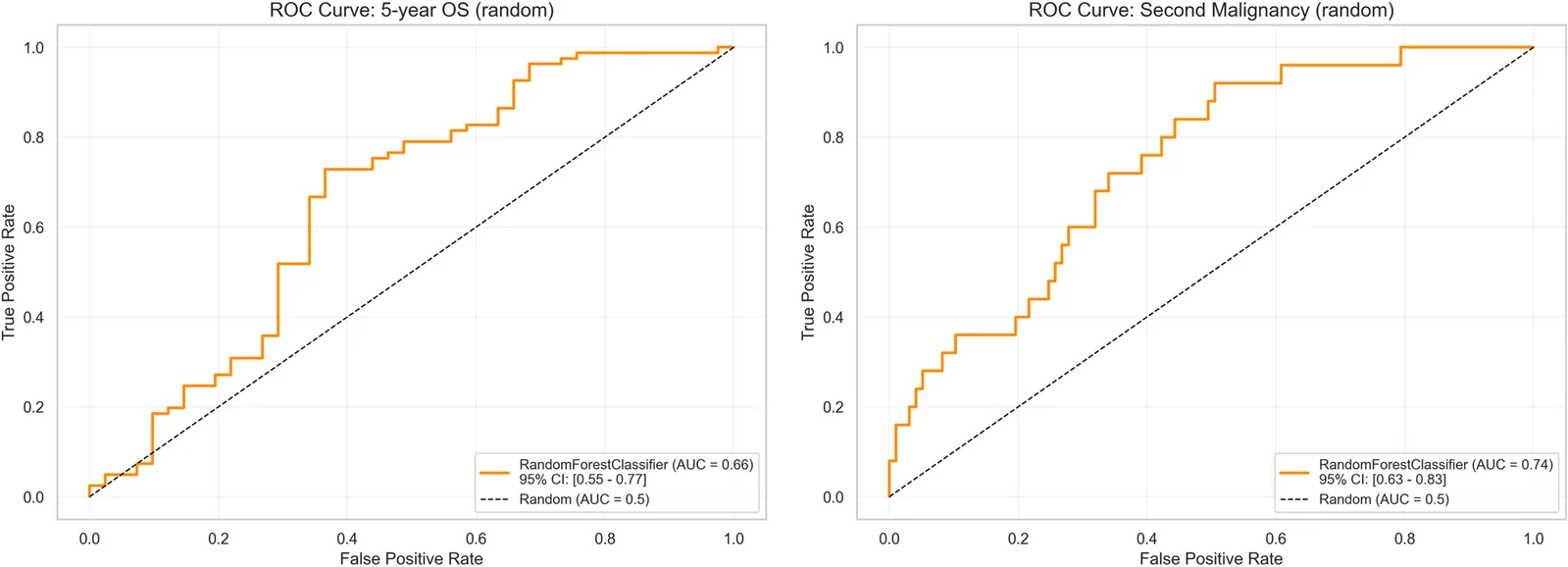
Area under the curve for Random Forest algorithm (random split with blood inflammatory markers) for prediction of 5-year OS and SPC

Best SPC prediction was obtained through random RF classifier, with all features (0.8033 accuracy in validation cohort with AUC: 0.7427. The model was optimized using 200 trees with a maximum depth of 10; in this setup, the algorithm allowed for more granular partitioning with a minimum of 2 samples required both for splitting a node and for defining a terminal leaf. Without blood inflammatory markers, random RF classifier obtained 0.7951 accuracy with AUC: 0.6237. The model achieved this performance with an architecture of 50 trees and a maximum depth of 5, requiring at least 2 samples for both node splitting and leaf definition. ROC-AUC curves for the best performing algorithms are depicted in Fig. 2. To evaluate the methodological replication of the machine learning boundaries, internal cross-validation experiments were executed, with k-cross-validation k = 3. For the configurations incorporating systemic inflammatory markers, the evaluation metrics demonstrated a high level of analytical consistency, confirming that the algorithmic performance does not rely on a specific partitioning of the dataset.The validation indices for the complete models remained stable, yielding a mean Accuracy of 0.67 for the 5-year OS prediction with blodd inflammatory markers included in the algorith, and 0.85 for the SPC risk assessment. These values successfully aligned with the parameters established during the primary independent testing phase, thereby verifying the reliability of the captured biological signal.

**Fig. 3:**
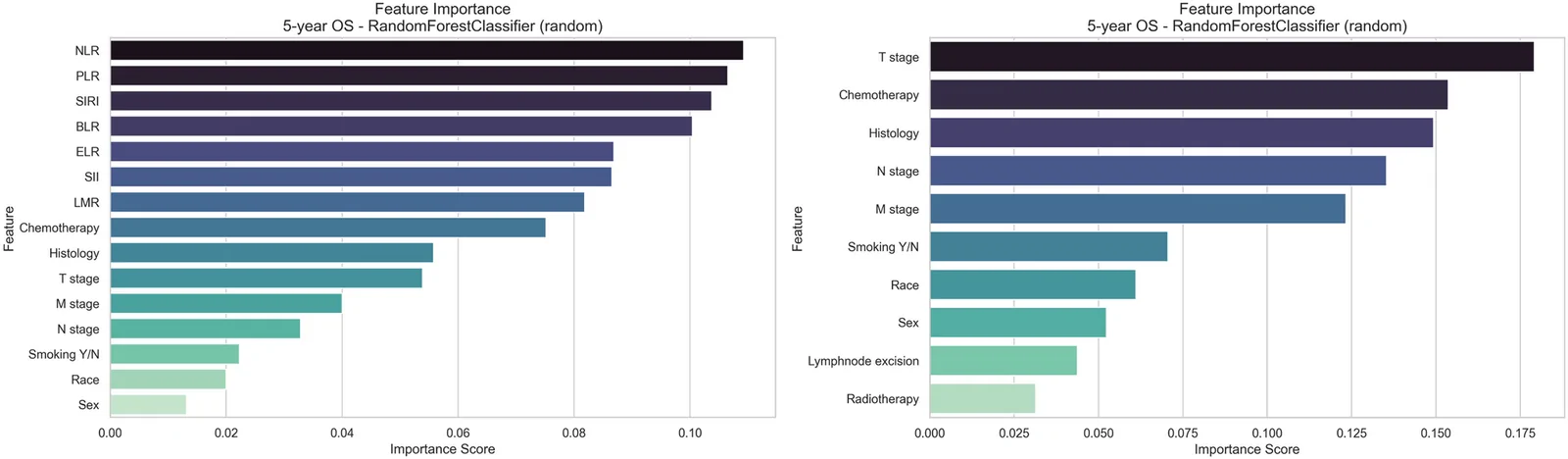
Feature importance for the Random Forest algorithm (random split) for prediction of 5-year OS, with and without blood inflammatory markers

**Fig. 4:**
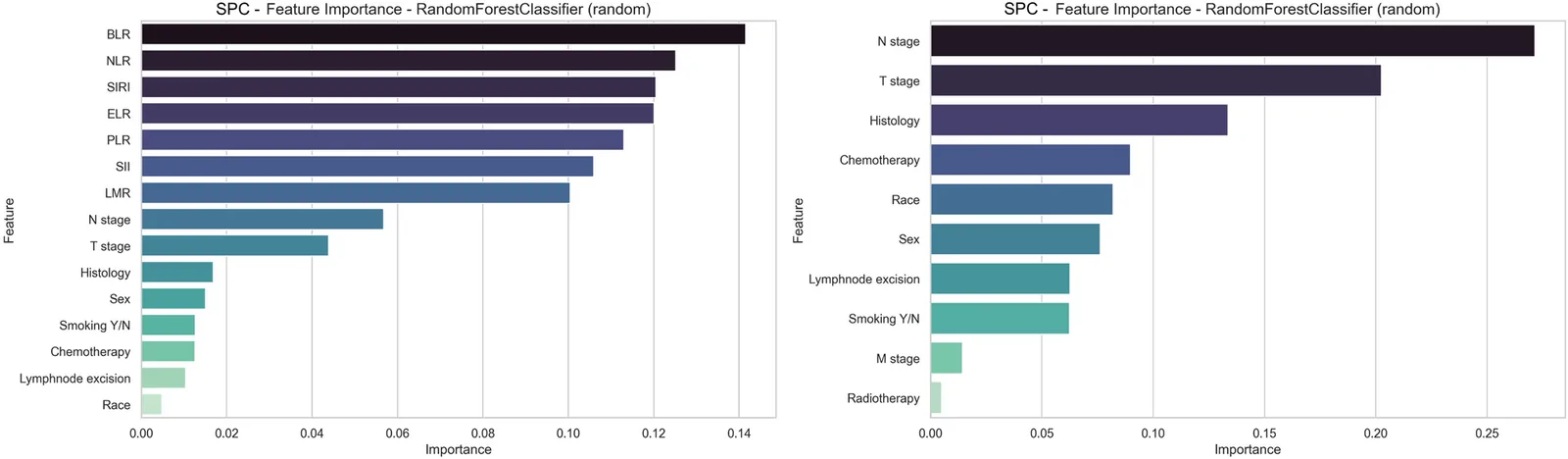
Feature importance for the Random Forest algorithm (random split) for prediction of SPC, with and without blood inflammatory markers SUPPLEMENT

We also evaluated and ranked the utility of each variable in the prediction algorithms. In prediction of 5-year overall survival, blood-derived inflammatory markers emerged as the most influential features within the machine-learning algorithm. These were followed, in descending order of importance, by receipt of chemotherapy, histological subtype, TNM stage, smoking status, race, and sex. When blood inflammatory markers were excluded from the model, T stage was the most important predictor, followed by chemotherapy, histology, N and M stage, smoking status, race, sex, lymph node excision, and radiotherapy regimen. Importance plots were generated for the most significant predictors of each high-performance algorithm with and without blood inflammatory markers, Fig. 3. For SPC prediction, the model incorporating all available features identified blood inflammatory markers as the most important predictors. These were followed by N and T stage, histological subtype, sex, smoking status, chemotherapy, lymph node excision, radiotherapy regimen and race, in descending order of feature importance. When inflammatory ratios were excluded from the model, N and T stage emerged as the most important predictors. These were followed by histological subtype, chemotherapy, race, sex, lymph node excision, smoking status, M stage, and, lastly, radiotherapy regimen. Graphs for features importance in SPC prediction are depicted in Fig. 4. The present study was reported according to Transparent Reporting of a Multivariable Prediction Model for Individual Prognosis or Diagnosis (TRIPOD + AI) reporting guidelines.

## Discussion

In this study, we applied established machine learning methods to develop prognostic models for 5-year OS and SPCs in a non-endemic multicentric European NPC population, using demographic, tumour-related, treatment-related, and pre-treatment systemic variables. Our results indicate that models incorporating blood-based inflammatory markers achieved the best predictive performance, with RF and SVM algorithms performing consistently across different data splits. Chronic inflammation plays a pivotal role in the initiation and progression of malignancies, predisposing individuals to the development of various cancer types [19, 20].

Multiple studies have investigated the prognostic value of systemic inflammatory markers in oncology, including head and neck cancer [21–25]. The systemic inflammation response index (SIRI), initially described by Qi et al. in pancreatic cancer [22], has been consistently associated with poorer overall survival in NPC and subsequently validated as an independent prognostic factor in NPC patients treated with IMRT [23–25]. Other inflammatory indices, including PLR, LMR, and SII, have likewise demonstrated prognostic value in NPC [26–28], with PLR shown to complement EBV-DNA–based risk stratification and SII serving as a prognostic biomarker in this setting. In addition, meta-analyses have further confirmed the prognostic ability of elevated NLR on OS, progression-free survival (PFS), disease-specific survival (DSS), and distant metastasis–free survival (DMFS) in NPC [29, 30]. Notably, high SII levels have also been associated with inferior 5-year OS, DMFS, and PFS [31]. A study from Wuhan involving 255 NPC patients demonstrated that elevated inflammatory indices, including SII, SIRI, NLR, and PLR, were associated with poorer OS, with combined markers outperforming individual haematological parameters in prognostic accuracy [3]. These findings suggest that composite inflammatory indices may complement TNM staging and improve risk stratification in NPC. Importantly, TNM stage variables were incorporated into all machine-learning models; therefore, the observed contribution of inflammatory markers and other clinicopathological features suggests a potential incremental prognostic value beyond TNM staging alone. Similarly, the Pan-Immune-Inflammation Value (PIV), calculated from pre-treatment blood results, was associated with significantly better OS, and PIV emerged as an independent prognostic factor alongside TNM stage and body mass index (BMI) [32].

Another emerging prognostic tool in oncology is the Glasgow Prognostic Score (GPS), which integrates serum C-reactive protein (CRP) and albumin levels. GPS has demonstrated prognostic relevance across multiple malignancies^33, 34,35^, including nasopharyngeal carcinoma (NPC)^36^. Nevertheless, as GPS partially reflects the nutritional and systemic inflammatory status of patients, its prognostic performance appears to be more pronounced in advanced-stage NPC. Furthermore, BMI has shown potential influence on the prognostic utility of GPS, and given the retrospective design of the present study, BMI data were not consistently available and therefore could not be incorporated into the analysis.

Clinical nomograms incorporating inflammatory blood markers have improved prognostic accuracy in NPC, achieving C-indices of up to 0.70 and AUC values of 0.74 for OS prediction [37, 38]. In pursuit of more accurate prognostic tools, recent studies have increasingly employed ML algorithms to enhance prediction of survival outcomes in NPC. AI has shown strong performance in predicting cancer survival and progression by capturing complex prognostic patterns within high-dimensional data [13]. Integrating radiomics with machine-learning techniques has further improved predictive accuracy, with reported performance metrics exceeding 0.80 in several studies [39–44]. A ML model developed in Zhejiang demonstrated superior predictive performance for post-treatment recurrence of NPC, compared with traditional Cox and competing-risk models [45]. Similarly, a recent Chinese study reported that a comprehensive ML model integrating clinicopathological, biological, and MRI features achieved higher 5-year AUCs for progression-free survival than models based solely on clinical data [12]. Despite these advances, clinical implementation of radiomics remains limited due to algorithmic heterogeneity, lack of standardized imaging and feature-extraction protocols, and substantial computational requirements associated with radiomics-based approaches [6, 46].

Our prediction algorithm achieved higher predictive accuracy than that reported by Zuo et al. [12], despite relying exclusively on clinical variables and excluding radiomics features. The comparable performance may partly reflect differences in predicted outcomes (OS vs. PFS), stage distribution, and patient ethnicity. Importantly, our model is readily applicable in routine clinical practice, as it does not require complex image processing. The present machine-learning algorithms demonstrated good predictive performance for both OS and SPC occurrence in NPC patients treated with IMRT, with further improvement observed when inflammatory markers were added to clinicopathological variables. This effect was particularly noticeable for SPC prediction, and feature-importance analyses identified inflammatory ratios among the most influential predictors for both outcomes. Chemotherapy also ranked highly for OS prediction, consistent with evidence demonstrating improved OS and PFS when combined with radiotherapy [8, 47–49]. Reported 5-year OS rates for NPC in literature range from 40% to 65% across combined stages [8, 47]; thus, the 66.6% 5-year OS observed in our cohort is satisfactory. Given the predominance of advanced-stage disease, the favourable survival outcomes may be partly attributed to the high proportion of patients (77.3%) receiving combined-modality treatment.

The relatively high importance of the histological subtype for 5-year survival prediction aligns with findings from a large review conducted in the United States, which underscored the significant influence of both histology and ethnicity on survival outcomes in NPC. Katelyn et al. [2] demonstrated both histological subtype and race were identified as significant determinants of survival in NPC. Patients with non-keratinizing, undifferentiated NPC exhibited the most favourable outcomes, whereas those with keratinizing histology had the poorest prognosis. The 5-year OS rates were 68.9% for non-keratinizing, undifferentiated NPC; 61.7% for non-keratinizing, differentiated NPC; and markedly lower at 42.8% for keratinizing NPC. When stratified by ethnicity, among patients with keratinizing NPC, Asian patients demonstrated the best 5-year OS (61.8%), followed by Hispanic patients (50.9%), whereas Caucasian (40.8%) and Black (37.7%) patients had comparably poor outcomes. In contrast, among patients with non-keratinizing differentiated NPC, Asians achieved the highest survival (5-year OS: 68.5%), while Caucasian patients had the lowest (58.0%). Our population included *a predominantly European non-endemic population*, and the most frequent histology was non-keratinizing undifferentiated (WHO III).

Smoking is a well-established risk factor for NPC, particularly among patients diagnosed after the age of 50 [2]. The mean age in our patient cohort is 52 years, and the percentage of smokers was relatively high (37.7%), factors that may partly account for the high incidence of SPC in our cohort (15.8%). The observed locations of SPC in our NPC patients are consistent with literature findings [4] and predominantly involve sites commonly affected by tobacco-related carcinogenesis.

The Surveillance, Epidemiology, and End Results (SEER) program covers approximately 30% of the U.S. population and has also been used to develop survival prediction models for NPC [5, 50]. Lin et al. [50] demonstrated superior survival in patients with advanced disease treated with combined radiotherapy and chemotherapy and developed ML models predicting 3- and 5-year OS, achieving C-indices of 0.73 and 0.75, respectively, comparable to those observed in our analysis. Similarly, Qiu et al. [5] applied a RF model to predict 3-year OS, reporting an accuracy of 0.94 and an AUC of 0.72 in the test cohort. The higher accuracy in their study likely reflects differences in patient selection, specifically the inclusion only of metastatic NPC cases, as well as the shorter prediction horizon (3-year vs. 5-year overall survival).

Studies investigating SPCs in NPC patients remain scarce and are largely derived from high-incidence regions [4, 16, 51]. A comprehensive review including 89,168 patients from 21 studies (1961–2017) reported a mean SPC incidence of 6.6% (range, 1.5–20.2%), with lower rates in high-incidence areas (4.9%) compared with low-incidence regions such as Europe and North America (8.7%) [4]. The most common SPC sites were the oral cavity, pharynx, sinonasal tract, oesophagus, and lung, with a mean latency of occurrence of approximately 7.7 years. To our knowledge, the present study is the first to apply machine-learning models, incorporating systemic inflammatory markers to predict SPC risk in NPC patients.

A major strength of this study is the inclusion of a *predominantly* European NPC cohort, with over 90% of patients being White, providing relevant insights into prognostic modelling in non-endemic regions. Most previous studies incorporating clinicopathological and inflammation-based predictive algorithms have been conducted in high-incidence Asian populations, where genetic, environmental, and biological factors may differ substantially. By analysing patients from a low-incidence European setting, our findings support the applicability of clinicopathological and systemic inflammatory markers for outcome prediction in contexts where EBV DNA testing and advanced radiomics are not routinely available. Although the sample size is limited due to the low incidence of NPC in Europe, the inclusion of patients with a minimum follow-up of five years enhances the robustness and clinical relevance of our results and provides a foundation for future studies.

Several limitations of this study should be acknowledged. First, its retrospective design is inherently associated with a risk of selection bias, and despite adjustment for known confounders, unmeasured factors may have influenced the results. No formal adjustment was made for potential confounders such as pre-existing comorbidities and patient body mass index (BMI) at the time of blood sampling, which may significantly influence systemic inflammatory markers. While this represents a potential threat to the validity of the predictive models, the consistency of ratios like NLR and SII as top predictors across multiple data splits suggests they capture a robust biological signal related to the tumour microenvironment rather than transient systemic noise. Moreover, the inclusion of multiple systemic inflammatory biomarkers that are biologically and mathematically interrelated, which may introduce multicollinearity and influence model stability and interpretability. Although the machine-learning algorithms used, particularly Random Forest models, are generally less sensitive to multicollinearity than conventional regression approaches, no formal collinearity reduction strategy or dimensionality analysis was performed. Consequently, some degree of redundancy between predictors may have affected feature importance estimates and overall model robustness. Future studies incorporating larger multicenter cohorts, external validation, and dedicated variable-selection or dimensionality-reduction techniques are warranted to further optimize prognostic model performance and generalizability. Another limitation of the current research is the absence of data regarding plasma EBV DNA for all patients and were therefore excluded from the analysis; subset modelling was not feasible due to limited sample size. As a result, we could not compare the prognostic value of EBV DNA with systemic inflammatory markers or incorporate it into the predictive algorithm, which might have improved its performance. Although EBV DNA has demonstrated diagnostic and prognostic relevance in NPC [52, 53], lack of standardization and inter-laboratory variability currently limit its widespread clinical applicability. Furthermore, although TNM variables were included in all prediction models, a formal comparison against a dedicated TNM-only model was not performed. Future studies with larger external cohorts should evaluate the incremental predictive value of machine-learning approaches over conventional staging systems alone. An additional limitation of the present study is the potential treatment heterogeneity introduced by the multicenter design. Although all participating institutions are tertiary oncological centers treating nasopharyngeal carcinoma according to internationally accepted guidelines, variations in treatment strategies, imaging protocols, laboratory assessments, supportive care, and follow-up practices may have influenced patient outcomes and model performance. Due to the retrospective nature of the study and the relatively limited sample size of some participating centers, formal center-effect or hierarchical analyses were not performed. Although efforts were made to harmonize clinicopathological and laboratory variables across institutions, residual inter-center variability cannot be excluded and may have affected the generalizability of the findings. Furthermore, the latency period of SPCs could not be calculated. Future integration of radiomics and biomolecular or genetic data may further enhance predictive performance and support more refined personalization of NPC management. While the predictive power of our algorithm is not yet optimal, it has demonstrated statistically meaningful predictive trends in a predominantly European cohort of patients. Therefore, these results should be interpreted as exploratory and hypothesis-generating rather than directly applicable to clinical decision-making. The present models are not intended to function as standalone prognostic tools, but rather to illustrate the potential complementary value of routinely available systemic inflammatory biomarkers in risk stratification for nasopharyngeal carcinoma. Further refinement through larger prospective cohorts, external validation, and integration of additional molecular, imaging, and treatment-related variables is necessary before clinical implementation can be considered.

## Supplementary Information

Below is the link to the electronic supplementary material.

Supplementary Material 1

## Data Availability

Anonymized data supporting the findings of this study are available from the corresponding author upon reasonable request, in accordance to institutional and ethical approval.
