# Supplementary material for: Machine learning prognostication in nasopharyngeal carcinoma: a european multicentre analysis of survival and risk of second malignancy
Source: Eur Arch Otorhinolaryngol. 2026 Jul 17;283(10):6651–63. doi: 10.1007/s00405-026-10461-z (PMC13615099; doi:10.1007/s00405-026-10461-z)
Supplement: Supplementary file 1 [file 405_2026_10461_MOESM1_ESM.docx]

**Table 4-** Cut-off values for blood inflammatory markers in each center

| Center name | SIRI | NLR | SII | PLR | LMR | ELR | BLR |
| --- | --- | --- | --- | --- | --- | --- | --- |
|  |  |  |  |  |  |  |  |
| UK-Cambridge | 1.744 | 3.002 | 942.814 | 183.919 | 3.6 | 83 | 28 |
| Spain | 2.866 | 3.821 | 1068.796 | 184.758 | 3.881 | 124 | 36 |
| Romania | 2.043 | 3.4 | 893.707 | 162.318 | 3.586 | 77 | 42 |
| UK- London | 1.937 | 3.564 | 1038.212 | 216.431 | 2.778 | 133 | 53 |
| Italy- Treviso | 1.621 | 2.996 | 650.408 | 161.191 | 3.364 | 134 | 21 |
| Italy- Brescia | 2.699 | 4.108 | 887.952 | 184.892 | 2.508 | 102 | 25 |

SIRI, systemic Inflammation Response Index; NLR, Neutrophil to Lymphocyte Ratio; SII, Systemic Immune-Inflammation Index; PLR, Platelet to Lymphocyte Ratio; LMR, Lymphocyte to Monocyte Ratio; ELR, Eosinophil to Lymphocyte Ratio; BLR, Basophil to Lymphocyte Ratio

**Table 5-** Prediction of 5 year OS through three different machine learning algorithms and with the study population split in three manners- stratified, random and balanced- results in training cohort

| **Prediction of 5-year OS - Training** | | | | | | | |
| --- | --- | --- | --- | --- | --- | --- | --- |
|  | **Model** | **Split Type** | **Accuracy** | **Precision** | **Recall** | **F1 Score** | **AUC** |
| Algorithms with blood  inflammatory markers | Decision Tree | stratified | 0,8410 | 0,8414 | 0,8410 | 0,8345 | 0,8444 |
|  | Random Forest | stratified | 0,9682 | 0,9689 | 0,9682 | 0,9679 | 0,9988 |
|  | SVM | stratified | 0,7456 | 0,7374 | 0,7456 | 0,7389 | 0,7877 |
|  | Decision Tree | random | 0,9187 | 0,9267 | 0,9187 | 0,9201 | 0,9822 |
|  | Random Forest | random | 0,8834 | 0,9007 | 0,8834 | 0,8757 | 0,9886 |
|  | SVM | random | 0,7809 | 0,7801 | 0,7809 | 0,7645 | 0,8378 |
|  | Decision Tree | balanced | 0,7660 | 0,7880 | 0,7660 | 0,7614 | 0,8035 |
|  | Random Forest | balanced | 0,8883 | 0,8883 | 0,8883 | 0,8883 | 0,9667 |
|  | SVM | balanced | 0,7500 | 0,7690 | 0,7500 | 0,7455 | 0,8358 |
| Algorithms without blood inflammatory markers | Decision Tree | stratified | 0,8516 | 0,8497 | 0,8516 | 0,8489 | 0,9198 |
|  | Random Forest | stratified | 0,8622 | 0,8705 | 0,8622 | 0,8547 | 0,9113 |
|  | SVM | stratified | 0,7809 | 0,7760 | 0,7809 | 0,7769 | 0,8026 |
|  | Decision Tree | random | 0,7986 | 0,7941 | 0,7986 | 0,7922 | 0,8679 |
|  | Random Forest | random | 0,7951 | 0,8079 | 0,7951 | 0,7749 | 0,8742 |
|  | SVM | random | 0,7809 | 0,7843 | 0,7809 | 0,7622 | 0,8108 |
|  | Decision Tree | balanced | 0,7368 | 0,7503 | 0,7368 | 0,7333 | 0,8020 |
|  | Random Forest | balanced | 0,7737 | 0,7774 | 0,7737 | 0,7729 | 0,8663 |
|  | SVM | balanced | 0,8053 | 0,8180 | 0,8053 | 0,8033 | 0,9071 |

OS, Overall Survival; SVM, Support Vector Machines; AUC, Area Under the Curve;

**Table 6-** Prediction of SPC through three different machine learning algorithms and with the study population split in three manners- stratified, random and balanced- results in training cohort

| **Prediction of Second Primary Cancer - Training** | | | | | | | |
| --- | --- | --- | --- | --- | --- | --- | --- |
|  | **Model** | **Split Type** | **Accuracy** | **Precision** | **Recall** | **F1 Score** | **AUC** |
| Algorithms with blood  inflammatory markers | Decision Tree | stratified | 0,9223 | 0,9191 | 0,9223 | 0,9200 | 0,9701 |
|  | Random Forest | stratified | 0,8622 | 0,8816 | 0,8622 | 0,8147 | 0,9777 |
|  | SVM | stratified | 0,7739 | 0,7232 | 0,7739 | 0,7461 | 0,4714 |
|  | Decision Tree | random | 0,9576 | 0,9569 | 0,9576 | 0,9555 | 0,9920 |
|  | Random Forest | random | 0,9576 | 0,9596 | 0,9576 | 0,9543 | 0,9999 |
|  | SVM | random | 0,8375 | 0,7979 | 0,8375 | 0,8126 | 0,2980 |
|  | Decision Tree | balanced | 0,8444 | 0,8814 | 0,8444 | 0,8406 | 0,9353 |
|  | Random Forest | balanced | 0,8778 | 0,8825 | 0,8778 | 0,8774 | 0,9768 |
|  | SVM | balanced | 0,5778 | 0,7160 | 0,5778 | 0,4974 | 0,2563 |
| Algorithms without blood  inflammatory markers | Decision Tree | stratified | 0,8622 | 0,8542 | 0,8622 | 0,8231 | 0,8700 |
|  | Random Forest | stratified | 0,8410 | 0,7073 | 0,8410 | 0,7684 | 0,8446 |
|  | SVM | stratified | 0,7845 | 0,7194 | 0,7845 | 0,7484 | 0,5197 |
|  | Decision Tree | random | 0,8693 | 0,8408 | 0,8693 | 0,8381 | 0,8761 |
|  | Random Forest | random | 0,8622 | 0,7434 | 0,8622 | 0,7984 | 0,8687 |
|  | SVM | random | 0,7986 | 0,7596 | 0,7986 | 0,7776 | 0,4956 |
|  | Decision Tree | balanced | 0,7667 | 0,8111 | 0,7667 | 0,7580 | 0,8760 |
|  | Random Forest | balanced | 0,7333 | 0,7352 | 0,7333 | 0,7328 | 0,8662 |
|  | SVM | balanced | 0,8556 | 0,8571 | 0,8556 | 0,8554 | 0,9427 |

SPC, Second Primary Tumour; SVM, Support Vector Machines; AUC, Area Under the Curve.
